# Are chest compressions safe for the patient reconstructed with sternal plates? Evaluating the safety of cardiopulmonary resuscitation using a human cadaveric model

**DOI:** 10.1186/1749-8090-5-64

**Published:** 2010-08-18

**Authors:** Douglas R McKay, Hosam F Fawzy, Kathryn M McKay, Romy Nitsch, James L Mahoney

**Affiliations:** 1 Department of Surgery, Queen's University, Kingston, Ontario, Canada; 2 Department of Cardiac Surgery, University of Toronto, Toronto, Ontario, Canada; 3 Oceanworks International, Burnaby, British Columbia, Canada; 4 Queen's University, Department of Obstetrics and Gynecology, Kingston, Ontario, Canada; 5 Department of Plastic Surgery, University of Toronto, Toronto, Ontario, Canada

## Abstract

**Background:**

Plate and screw fixation is a recent addition to the sternal wound treatment armamentarium. Patients undergoing cardiac and major vascular surgery have a higher risk of postoperative arrest than other elective patients. Those who undergo sternotomy for either cardiac or major vascular procedures are at a higher risk of postoperative arrest. Sternal plate design allows quick access to the mediastinum facilitating open cardiac massage, but chest compressions are the mainstay of re-establishing cardiac output in the event of arrest. The response of sternal plates and the chest wall to compressions when plated has not been studied. The safety of performing this maneuver is unknown. This study intends to demonstrate compressions are safe after sternal plating.

**Methods:**

We investigated the effect of chest compressions on the plated sternum using a human cadaveric model. Cadavers were plated, an arrest was simulated, and an experienced physician performed a simulated resuscitation. Intrathoracic pressure was monitored throughout to ensure the plates encountered an appropriate degree of force. The hardware and viscera were evaluated for failure and trauma respectively.

**Results:**

No hardware failure or obvious visceral trauma was observed. Rib fractures beyond the boundaries of the plates were noted but the incidence was comparable to control and to the fracture incidence after resuscitation previously cited in the literature.

**Conclusions:**

From this work we believe chest compressions are safe for the patient with sternal plates when proper plating technique is used. We advocate the use of this life-saving maneuver as part of an ACLS resuscitation in the event of an arrest for rapidly re-establishing circulation.

## Background

Chest compressions are a cornerstone of cardiopulmonary resuscitation. Recent work confirms the importance of early compressions to improve survival [1]. Oxygen is present in the blood up to ten minutes after arrest; re-establishing circulation of this blood via sternal compressions is the most important step of the ABCs early in resuscitation [2].

Sternal wound dehiscence after median sternotomy can be a devastating complication. The mainstay of treatment has been aggressive debridement followed by flap closure. This diminishes mechanical chest wall integrity. A new advance, sternal repair with plate and screw fixation, can obviate the complications of persistent sternal instability. These include chronic pain, paradoxical chest wall motion, and decreased pulmonary function [3]. The modality is safe when used appropriately and confers the advantages of early extubation, tension-free repair and simple soft tissue advancements in lieu of more complicated flaps whilst restoring mechanical stability [4].

Cardiac or major vascular surgery places patients at a higher risk for perioperative cardiac events, and the subset whose wounds dehisce are typically at higher risk on the basis of medical comorbidity [5,6]. Some of this population will require perioperative resuscitation. The response of sternal plates and the plated chest wall to compressions has not been studied. Potential hypothesized pitfalls include hardware failure or skeletal and visceral trauma.

To determine the safety of performing this potentially life-saving maneuver, we designed an experiment to study the effects of chest compressions on sternal hardware and the thorax. We studied these outcomes using a human cadaveric model while monitoring intrathoracic pressure during a simulated resuscitation.

## Methods

Institutional Review Board ethics approval was applied for and granted for this study by the University of Toronto Ethics Review Office, protocol reference # 18535.

Compressions were performed on an un-plated cadaver to serve as control. Intrathoracic pressures were monitored in the control with the intrathoracic pressure monitoring system detailed below, placed inferior to the sternum through an incision in the diaphragm. No sternotomy was performed on the control experiment. The anterior thorax was exposed and checked for fracture. Observations were documented. In the experimental group, a midline sternotomy was performed on five fresh frozen cadavers. Bilateral composite myocutaneous pectoralis major flaps were elevated exposing the anterior thorax for plating.

A digital manometer that records pressure within a closed system at appropriate range and intervals was selected (Reed PM9100^®^, Alaron Instruments, Newmarket, ON). A 250 cc silicone bladder measuring 10 cm across was connected via fill tube and intravenous tubing to the manometer to create a closed system. This bladder was then seated immediately deep to the inferior third of the sternum and distended with air to conform to the cavity in which it was placed. (Mentor Corp., Santa Barbara, CA). The manometer was connected via RS232 cable to a laptop and configured to display real-time intrathoracic pressure while simultaneously recording absolute values, both in mmHg, every two seconds (SW-U801 for Windows^®^, Alaron Instruments, Newmarket, ON, Canada).

**Figure 1:**
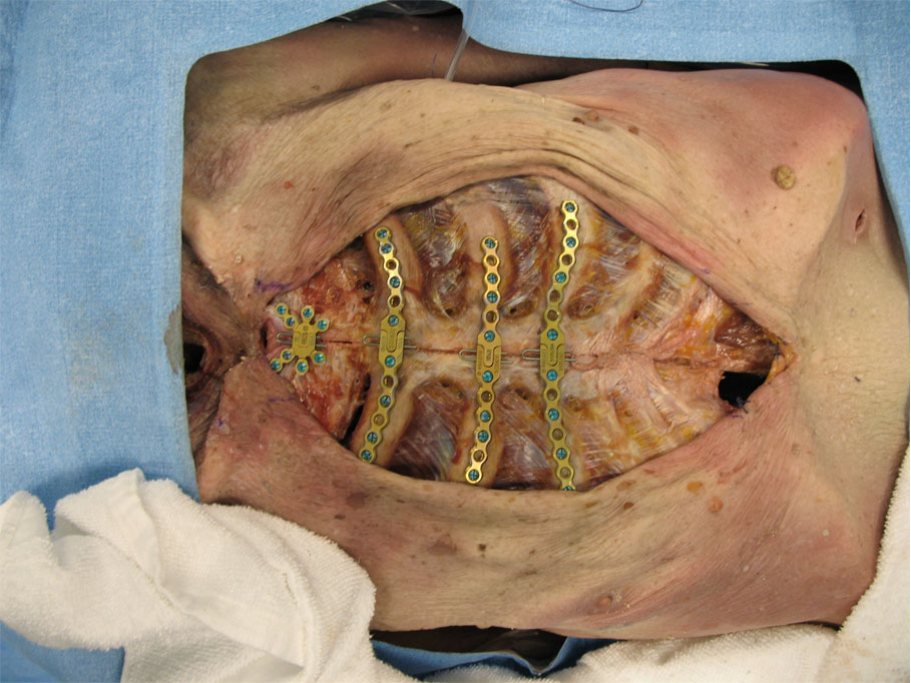
**Plated sternum**.

The sternum was reduced and held with forceps. The cadaver was plated using three rib plates combined with a single manubrial plate (See Fig. 1). Rib plates were placed on the second, third and fourth ribs (Titanium Sternal Fixation System, Synthes, USA). Holes were drilled using the system guide. A depth gauge was used to select the appropriate screw length. Our intent was that screws would catch the deep cortex without significantly breaching the cortex.

The incisions were closed in a layered fashion. Vicryl 2.0 sutures (Johnson & Johnson, Piscataway, NJ) were used for the deep layer and the skin was closed with skin staples (3 M, St. Paul, MN). The manometer was zeroed. A physician trained and experienced in performing cardiopulmonary resuscitation carried out compressions for a total of five minutes at a rate of 60 to 80 compressions per minute, on both the control and cadaver specimens. Intrathoracic pressures were displayed to the physician performing the resuscitation and chest compressions were maintained at a depth that generated minimum peak intrathoracic pressures of 60 mmHg.

The incisions were opened and the hardware and thorax were examined for trauma. Observations were recorded and photo-documented. An oscillating saw was used to completely excise the anterior thorax. The deep surfaces of the skeletal thorax and the viscera were examined for trauma. The plates and screws were removed. Each screw was removed from the plates and each plate was disassembled. Each screw, pin and plate was examined for damage or failure.

## Results

**Figure 2:**
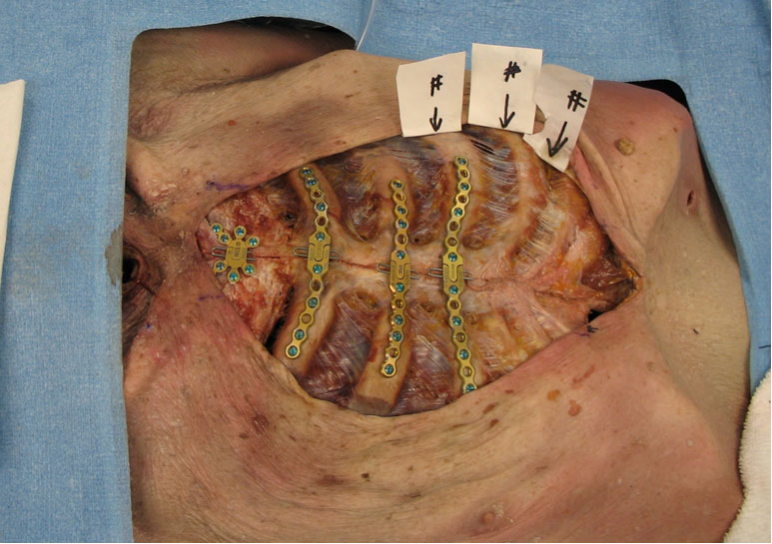
**Rib fractures after resuscitation**.

The plating mechanism was visually evaluated for damage and checked for functional compromise. No screw, pin or plate damage, or failure was noted. All pins and screws were removed with ease. All plates easily disengaged at the midline; there was no compromise of the mediastinal access mechanism secondary to the sustained compressions. No obvious pleural or visceral damage was noted. No rib fractures were noted in the plated zone. Rib fractures were noted in all cadavers beyond the limits of the sternal plates. (See Fig. 2) Two fractures were noted in a control specimen after an identical compression sequence (See Table 1). We were unable to physically generate a force that fractured the hardware.

**Table 1:** Rib fracture incidence and position relative to plated sternum; comparison between control and cadaveric specimens.

Specimen	Number of fractures	Location of fractures
		

Control	2	lateral, xyphoid

Cadaver 1	1	inferolateral

Cadaver 2	2	lateral, xyphoid

Cadaver 3	2	lateral

Cadaver 4	3	lateral

Cadaver 5	1	lateral

## Discussion

Predicting the risk of perioperative cardiac events is a complicated science. Patient risk estimates are based on a number of known risk factors. Cardiac and major vascular surgery places a patient at a higher risk for perioperative cardiac events and is highest for coronary artery surgery [7]. Those who dehisce sternotomy wounds may do so as the result of medical comorbidity. It follows that plated patients are more likely to arrest and require resuscitation. The safety of performing chest compressions in this group merits investigation.

Chest compressions are a traumatic procedure. Rib fracture is the most common complication. In a recent review, Hoke et al. summarize the literature on skeletal injury as a result of chest compression and discover a spectrum of fracture incidence in resuscitated adults ranging from 12.9% to 96.6% [8]. The most common complication of rib fractures is pain; pain may inhibit deep breathing, which may increase the risk of atelectasis or pneumonia. Despite the potential morbidity, they emphasize that the value of chest compressions outweigh the risk of skeletal damage and conclude that the risk of fracture should not deter an adequate and appropriate cardiopulmonary resuscitation in the event of arrest.

In our model, the plates appear to bolster the chest wall and prevent fracture immediately deep to the plated thorax. Our fracture incidence is higher than in the literature but consistent with our control. The observed fractures were all significantly beyond the plates and predominantly immediately lateral to the plates on the plated ribs. The incidence of rib fracture may be higher when compressions are performed on the plated sternum. This high incidence we observed may be a result of the frailty of the elderly cadaveric model, when compared to the documented incidence in the living.

One hypothesized source of morbidity was hardware failure and its potential to damage underlying viscera under dynamic compression. No hardware fracture or loosening was noted for either plates or screws. All were removed and examined individually. Both appear capable of enduring the dynamic stresses and absolute pressures encountered during resuscitation. We were unable to physically apply forces via compressions that resulted in hardware failure.

**Figure 3:**
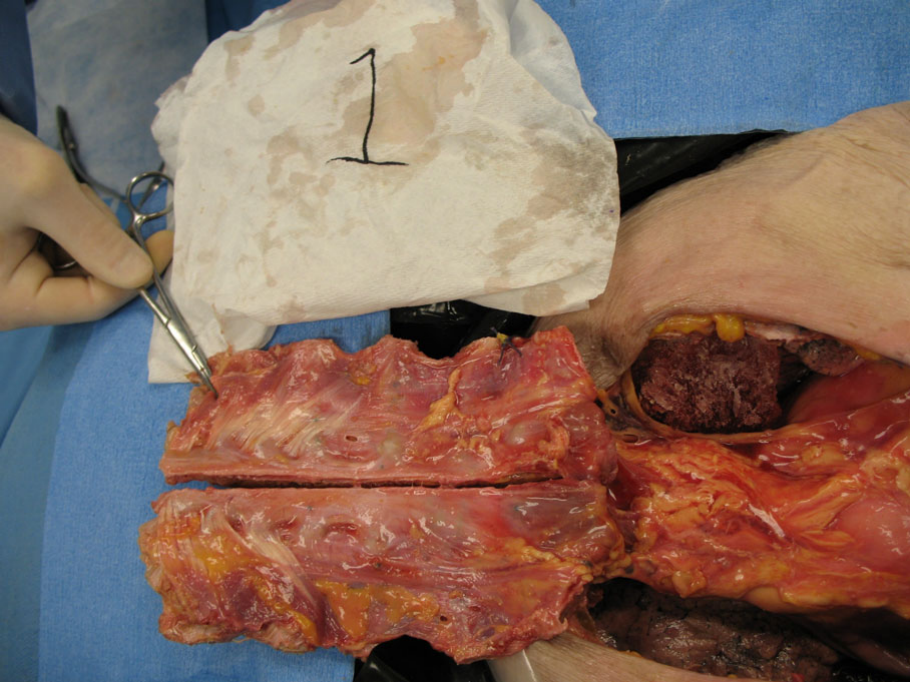
**Elevation and examination of deep sternal cortex and viscera**.

When the anterior thorax was removed and the cadaver examined, no obvious visceral trauma was noted (see Fig. 3). The screw depth appeared appropriate; none sat proud. Screws protruding from the inferior cortex may cause significant damage. We cannot over-emphasize the importance of proper screw selection when plating. At our institution we use preoperative CT scanning and measure and map absolute rib depth to ensure appropriate screw selection.

In the living, the adequacy of chest compressions has been measured via end-tidal CO2 levels, depth of compression and intra-thoracic pressure measurement. End tidal CO2 is the most commonly used modality. The cadaveric model is most amenable to intrathoracic pressure measurement.

Peak aortic compression pressures of 61 ± 22 mmHg have been measured via cook catheter during resuscitations in humans when performed by individuals experienced in cardiopulmonary resuscitation [9]. In order to ensure the plated sternum experienced an appropriate and adequate compressive force, a pressure manometer was attached to a closed bladder, and was inserted immediately deep to the sternum. The absolute pressure in this closed system was recorded every 2 seconds during resuscitation and displayed to the physician performing the compressions via digital readout. The compression depth was maintained to create a minimum peak intrathoracic pressure of 60 mmHg to accurately simulate mechanical forces experienced during the resuscitation. The maximum recorded pressure was 87 mmHg. There is a potential for slight inaccuracies in the absolute pressure measurements recorded.

This model has limitations. The distensible nature and elasticity of the silicone shell, fill tube, and intravenous tubing have the potential to alter pressure readings. Presumably this would result in a reading that was lower than the absolute pressure at peak and during decompression. Either scenario would mean the hardware was experiencing higher pressures than recorded. The silicone shell when distended and placed deep to the sternum has the potential to damage the underlying viscera but may also be protective.

The fresh frozen cadaveric model may not mimic the dynamics in the living. The frailty of the frozen and thawed cadavers may mislead us with regard to the true fracture incidence. We were unable to procure fresh cadavers; the use of fresh cadavers could significantly improve this study. The cost of procuring and preparing the cadavers limited the number of specimens used in the study and the power may be inadequate.

If screws are protruding deep to the deep cortex, compressions have the potential to inflict significant damage. Perioperative hypocoagulation may exacerbate potential complications. The risk of excessive screw length causing trauma during compressions may justify a postoperative CT. A significant breech of the deep cortex may modify recommendations to ward staff in the event of an arrest. One patient has arrested and undergone chest compressions after plating without adverse clinical sequelae.

## Conclusions

Based on our work with this human cadaveric model we believe chest compressions are safe in the plated sternum in the event of arrest with the caveat that appropriate screw length must be chosen. Chest compressions can be used to immediately re-establish blood flow and temporize until the chest may be re-opened according to the accepted algorithm for resuscitation after cardiac surgery. No hardware failure was observed. Rib fracture incidence beyond plates was higher than in the literature but comparable to control. Skeletal injury is well documented after chest compressions but fracture should not deter first responders from using chest compressions to re-establish circulation. This is also true for the plated patient.

## Competing interests

The authors declare that they have no competing interests.

## Authors' contributions

DRM wrote the grant and applied for funding, coordinated and executed the cadaveric study, analyzed the results and wrote the manuscript. HSM performed the sternotomy and plated the cadavers. KMM designed the pressure monitoring device used in the study. RN participated in the execution of the cadaveric resuscitation and plating study. JLM conceived of the study, participated in its execution and reviewed and edited the manuscript. All authors read and approved the final manuscript.
